# Psychological distress and the COVID-19 pandemic: the role of personality and coping strategies

**DOI:** 10.1590/0102-311XEN096123

**Published:** 2025-01-13

**Authors:** Sónia Catarina Carvalho Simões, Laura Maria Carreira Marques, Diogo André Fonseca Sequeira de Andrade, Sandra Isabel Ferreira das Neves Henriques, Luís André Abreu Paraíso Ferreira, Helena Maria Amaral do Espírito-Santo

**Affiliations:** 1 Instituto Superior Miguel Torga, Coimbra, Portugal.

**Keywords:** COVID-19, Psychological Distress, Coping Strategies, Personality, COVID-19, Angústia Psicológica, Estratégias de Enfrentamento, Personalidade, COVID-19, Distrés Psicológico, Estratégias de Enfrentamiento, Personalidad

## Abstract

Personality traits and coping strategies significantly predict predisposition to psychopathology. This study aimed to examine the predictive role of coping strategies in psychological distress during the COVID-19 pandemic in a sample of Portuguese individuals, considering personality and sociodemographic variables. Data were collected using Google Forms from 2402 individuals (86.8% women; mean age ± SD = 36.80 ± 11.80) between March and June 2020, found primarily through Facebook. The evaluation instruments included the *Brief Symptom Inventory* (BSI), *NEO Five-Factor Inventory*, and Brief-COPE. Younger adults, females, single individuals, and those with lower education experienced higher distress. Neuroticism was strongly associated with all dimensions of psychological distress and the overall BSI. Maladaptive coping strategies (self-distraction, denial, self-blame, behavioral disengagement) were positively correlated with distress, whereas agreeableness and positive reframing were negatively correlated. Regression analysis showed that gender, age, education, and psychiatric diagnosis predicted 12% of distress; adding neuroticism increased prediction to 34% and coping strategies to 37%, with self-blame among coping strategies being the strongest predictor. Personality traits and coping strategies were significant predictors of psychological distress during the COVID-19 pandemic. These findings emphasize the need for interventions that target neuroticism and maladaptive coping strategies to improve mental health outcomes during public crises.

## Abstract (pt)

Traços de personalidade e estratégias de enfrentamento predizem significativamente a predisposição à psicopatologia. Este estudo buscou examinar o papel preditivo das estratégias de enfrentamento ao sofrimento mental durante a pandemia da COVID-19 em uma amostra portuguesa considerando variáveis sociodemográficas e de personalidade. Os dados de 2.402 indivíduos (86,8% mulheres; Mage ± SD = 36,80 ± 11,80) foram coletados pelo Google Forms entre março e junho de 2020, majoritariamente através do Facebook. Os instrumentos incluíram o *Inventário Breve de Sintomas* (BSI, acronimo em inglês), o *Inventário de Personalidade de Cinco Fatores NEO* e o Brief-COPE. Jovens adultos, mulheres, indivíduos solteiros e aqueles com menor escolaridade experimentaram maior sofrimento. O neuroticismo foi fortemente associado a todas as dimensões do sofrimento mental e ao BSI geral. Estratégias de enfrentamento desadaptativas (autodistração, negação, autoculpa e desengajamento comportamental) foram positivamente correlacionadas com sofrimento, enquanto a agradabilidade e a ressignificação positiva foram negativamente correlacionadas. A análise de regressão mostrou que gênero, idade, escolaridade e diagnóstico psiquiátrico previram 12% do sofrimento, ao passo que adicionar neuroticismo à análise aumentou a previsão para 34% e estratégias de enfrentamento, para 37%. No mais, a autoculpabilização foi o preditor mais forte entre as estratégias de enfrentamento. Traços de personalidade e estratégias de enfrentamento foram preditores significativos de sofrimento mental durante a pandemia da COVID-19. Esses achados enfatizam a necessidade de intervenções que visem o neuroticismo e estratégias de enfrentamento desadaptativas para melhorar os desfechos de saúde mental durante crises públicas.

## Abstract (es)

Los rasgos de personalidad y las estrategias de afrontamiento predicen significativamente la predisposición a la psicopatología. Este estudio buscó examinar el papel predictivo de las estrategias para afrontar el sufrimiento mental durante la pandemia de COVID-19 en una muestra portuguesa considerando variables sociodemográficas y de personalidad. Se recopilaron datos de 2.402 personas (86,8% mujeres; Mage ± SD = 36,80 ± 11,80) por medio de Google Forms entre marzo y junio del 2020, principalmente por medio de Facebook. Los instrumentos incluyeron el *Inventario Breve de Síntomas* (BSI), el *Inventario de Personalidad de Cinco Factores NEO* y el Brief-COPE. Los adultos jóvenes, las mujeres, los solteros y aquellos con menor educación experimentaron un mayor sufrimiento. El neuroticismo se asoció firmemente con todas las dimensiones de la angustia mental y el BSI en general. Las estrategias de afrontamiento desadaptativas (autodistracción, negación, culpa y desvinculación conductual) se correlacionaron positivamente con el sufrimiento, mientras que la agradabilidad y el resignificación positiva se correlacionaron negativamente. El análisis de regresión mostró que el género, la edad, la educación y el diagnóstico psiquiátrico predijeron el 12% de la angustia, mientras que agregar el neuroticismo al análisis aumentó la predicción al 34% y las estrategias de afrontamiento al 37%. Además, la autoculpabilidad fue el predictor más fuerte entre las estrategias de afrontamiento. Los rasgos de personalidad y las estrategias de afrontamiento fueron predictores importantes de angustia mental durante la pandemia de COVID-19. Estos hallazgos ponen de manifiesto la necesidad de intervenciones dirigidas al neuroticismo y estrategias de afrontamiento desadaptativas para mejorar los resultados de salud mental durante las crisis públicas.

## Introduction

The year 2020 was marked by COVID-19, recognized as a pandemic due to its rapid spread worldwide. To halt it, quarantine, isolation, and social distancing measures were enforced to reduce social interactions ^1^ ^,^ ^2^. It has been found, however, that social isolation and the instability caused by the COVID-19 pandemic negatively affected mental health ^3^ ^,^ ^4^. High contagion rates, fear, and vulnerability to severe illness and death, along with the lack of knowledge about the disease ^5^ and social isolation, led to significant psychological impacts, including symptoms of anxiety and depression, sleep problems, stress, fear, guilt, sadness, helplessness, post-traumatic stress, and use of psychoactive substances, particularly among young adults under 35 years of age ^3^ ^,^ ^6^ ^,^ ^7^.

Various COVID-19-related risks and protective factors have been highlighted. Risk factors for psychological distress included being women, younger age, having a low socioeconomic or educational status, having a higher risk of contracting the disease, being under social isolation, and being a health worker, especially frontline ^8^ ^,^ ^9^ ^,^ ^10^ ^,^ ^11^ ^,^ ^12^ ^,^ ^13^ ^,^ ^14^ ^,^ ^15^ ^,^ ^16^ ^,^ ^17^ ^,^ ^18^. In contrast, access to medical resources, updated information on the disease, and the employment of protective measures have been cited as protective factors ^7^. Earlier studies had already highlighted symptoms of anxiety, aggression, and anger in individuals in social isolation due to the Middle East Respiratory Syndrome, which could lead to post-traumatic stress without early intervention ^19^.

Personality has been shown to be a relevant predictor for predisposition to develop psychopathology ^20^ and adherence to government policies during a pandemic. Specifically, individuals with higher neuroticism tend to avoid danger ^21^, making them more likely to comply with rules and feel safe ^22^. Similarly, those with higher agreeableness comply with rules due to their prosocial functioning ^23^, as their behavior aims to protect others ^22^. Individuals with greater openness to experiences often engage in artistic and intellectual expressions ^21^, which can protect mental health. People with higher conscientiousness, who value cleanliness and order ^24^, may find social isolation less disruptive ^25^. Conversely, those with higher extroversion, needing interpersonal contact and mobility, may find isolation challenging ^22^. Extroversion and conscientiousness influence compliance with COVID-19 prevention measures: higher extroversion leads to less social distancing, while higher conscientiousness leads to greater adherence to social distancing and norms ^26^.

The COVID-19 pandemic was, in fact, stressful for the entire world population, and each person used coping/confrontation strategies to deal with the situation. In addition, coping strategies and personality are among the most important predictors of psychopathology, accounting for 40% to 50% of the variance, with conscientiousness, extraversion, and neuroticism being the best psychopathology predictors ^27^. Furthermore, in a study by Knoll et al. ^28^, coping strategies were a more reliable predictor of stress when compared to personality.

Some inconsistencies in COVID-19 knowledge have fostered the emergence of negative emotions that could quickly evolve into the psychopathological conditions described above ^5^ ^,^ ^29^. To be able to work on the prevention/treatment of psychological consequences, it is pertinent to enrich the research related to the predictors of psychological distress during the COVID-19 pandemic. Therefore, we administered a set of questionnaires designed to explore various aspects that occurred during the timeframe coinciding with the COVID-19 pandemic.

The present study aimed to examine the relationships between sociodemographic and clinical variables, personality traits, coping strategies, and psychological distress during the COVID-19 pandemic. Specifically, we aimed to:

(1) Compare psychological distress levels across different sociodemographic and clinical groups (gender, age, educational level, profession, and having a psychiatric diagnosis);

(2) Examine the correlations between psychological distress and personality traits (neuroticism and agreeableness), as well as coping strategies (denial, self-blame, self-distraction, positive reinterpretation, and behavioral disengagement);

(3) Predict psychological distress using sociodemographic variables, personality traits, and coping strategies.

Based on these objectives, we formulated the following hypotheses:

(1) Higher levels of psychological distress will be observed among women, younger individuals, those with less formal education, and those with a psychiatric diagnosis;

(2) Psychological distress will be significantly correlated with personality traits (higher neuroticism and lower agreeableness) and coping strategies (greater use of denial, self-blame, and behavioral disengagement; lesser use of self-distraction and positive reinterpretation);

(3) Personality traits (neuroticism and agreeableness) and coping strategies (denial, self-blame, self-distraction, positive reinterpretation, and behavioral disengagement) will be significant predictors of psychological distress, even after controlling for sociodemographic variables.

## Method

### Participants

The inclusion criteria were individuals aged 18 years or older and of Portuguese nationality. The current study sample comprised 2,402 participants. The age range of the participants was 18-61, with a mean age of 36.80 years (standard deviation [SD] = 11.80 years). Table 1 illustrates the other sociodemographic and clinical characteristics. Some variables were recoded and grouped for subsequent analyses.

**Table 1:** Sociodemographic and clinical variables and comparison of the BSI-PSDI scores by groups (N = 2,402).

Variables/Groups	n	%	BSI-PSDI			
			M	SD	t/F	g/η^2^
Age (years)					17.37 *	.01
18-24	431	18.0	1.84	0.69		
25-50	1,658	69.0	1.72	0.62		
51 or more	312	13.0	1.57	0.53		
Gender					4.64 *	0.28
Female	2,086	86.8	1.75	0.63		
Male	316	13.2	1.57	0.59		
Marital status					9.54 *	0.01
Single	1,028	42.8	1.79	0.65		
Married	1,151	47.9	1.67	0.59		
Divorced/Separated/Widowed	223	9.3	1.71	0.63		
Formal education					5.42 *	0.01
Elementary School	93	3.9	1.81	0.73		
Secondary School	584	24.3	1.79	0.67		
Bachelor, Master’s and PhD	1,725	71.8	1.70	0.60		
Profession					3.52 **	0.01
Health & Service Care Workers	628	26.1	1.65	0.57		
Administrative, Managerial & Commerce Workers	749	31.2	1.71	0.62		
Education & Technical Professionals	763	31.8	1.78	0.64		
Manufacturing & Service Workers	114	4.7	1.78	0.71		
Law Enforcement, Legal, & Security	54	2.2	1.77	0.65		
Other	94	3.9	1.75	0.62		
Psychiatric disorder					15.83 **	0.94
No	2,073	86.3	1.65	0.58		
Yes	329	13.7	2.21	0.70		

BSI-PSDI: *Positive Symptom Distress Index of the Brief Symptom Inventory*; g = Hedge’s g effect size; η^2^: sum of squares between groups/total sum of squares; M: mean; SD: standard deviation.

Note: higher percentages and statistically significant higher means are in bold.

* p < 0.05;

** p < 0.01.

### Procedures

We used a non-probabilistic convenience sampling approach employing the snowball technique and sharing the study on social networks. This study was approved and shared by the Portuguese Association of Psychologists (06/May/2020), within the scope of the Scientific research support in psychological health and behavioral change in the COVID-19 pandemic sector. The study also received approval from the Ethics Committee of the Miguel Torga Institute of Higher Education (CE-P04-22).

We used Google Forms to collect data due to its ease of access and distribution, following the *Checklist for Reporting Results of Internet E-Surveys* (CHERRIES) guidelines ^30^:

(1) Development and pre-testing: the survey was pre-tested with a small group of individuals to ensure clarity and functionality.

(2) Recruitment process and sample access: the survey link was posted on Facebook in targeted groups, including health and university groups. Given these groups’ nature, most members were over 18 years old. The survey description also explicitly stated the age requirement to ensure compliance.

(3) Consent: participants received detailed information and provided consent electronically before proceeding.

(4) Data protection: anonymity and confidentiality were guaranteed, with data used solely for research.

(5) Survey administration: participants could complete the survey at their own pace and exit at any time. Data collection took place between 20 March and 29 June 2020.

(6) Response rate calculation: we did not track the number of clicks on the survey link due to the organic nature of distribution on Facebook.

(7) Preventing multiple entries from the same individual: duplicate entries were screened by cross-referencing demographic information and response patterns. No duplicate responses were found.

(8) Data analysis: the data were cleaned and analyzed using appropriate statistical methods to ensure accuracy and reliability.

All procedures performed in studies involving human participants were per the ethical standards of the Miguel Torga Higher Education Institute Ethics Committee and with the 1964 *Helsinki Declaration* and its later amendments or comparable ethical standards.

### Instruments

To characterize participants, a sociodemographic and clinical questionnaire included questions on age, gender, marital status, formal education, profession, and having/not having a psychiatric disorder diagnosis. Professions were categorized into health & service care workers (e.g., Physician, Nurse, Pharmacy technician, Psychologist, Radiology technician, Social worker), administrative, managerial, & commerce workers (e.g., administrator, manager, salesperson, travel agent), education & technical professionals (e.g., engineer, journalist, teacher, student, university professor), manufacturing & service workers (e.g., cleaning staff, factory worker, kitchen assistant), law enforcement, legal, & security (e.g., lawyer, military, police officer), and other (e.g., retired, unemployed). The psychiatric disorder diagnosis was self-reported and based on the current diagnosis status.

We used the *Brief Symptom Inventory* (BSI) ^31^ ^,^ ^32^, a 53-item questionnaire, to assess psychological distress. The BSI evaluates psychopathological symptomatology across nine dimensions (somatization, obsession-compulsive, anxiety, interpersonal sensitivity, depression, hostility, phobic anxiety, paranoid ideation, and psychoticism) and three global indexes. Each item is answered using a five-point Likert scale ranging from 0 (not at all) to 4 (extremely) considering a seven-day timeframe. Our study analyzed the nine dimensions and the *Positive Symptom Distress Index* (PSDI = the sum of the values of the items receiving non-zero responses/*Positive Symptom Total Index*) ^32^. The dependent variable was psychological distress (BSI-PSDI). Cronbach’s alpha of the nine dimensions ranged from 0.7-0.85 in the original study ^31^, 0.62-0.80 in the Portuguese study ^32^, and 0.75-0.88 in the present study.

The *NEO-Five Factor Inventory* ^33^ (NEO-FFI, Portuguese version ^34^) is a 60-item questionnaire that assesses the major five personality traits (with 11 items each), answered using a five-point Likert scale ranging from 0 (strongly disagree) to 4 (strongly agree). Cronbach’s alphas for the subscales used were adequate in the Portuguese adaptation version and the present study (respectively): conscientiousness (α = 0.81, α = 0.80), neuroticism (α = 0.81, α = 0.85), extraversion (α = 0.75, α = 0.75), agreeableness (α = 0.72, α = 0.65), openness to experience (α = 0.71, α = 0.64). NEO-FFI subscales were independent variables.

The *Brief-COPE Inventory* ^35^ (BCI, Portuguese version ^36^) is a 28-item questionnaire that assesses 14 coping strategies with two items for each strategy/subscale. The coping strategies include self-distraction, active coping, denial, substance use, use of emotional support, use of instrumental support, behavioral disengagement, venting, positive reframing, planning, humor, acceptance, religion, and self-blame. Each item is answered on a four-point Likert scale ranging from 0 (*I never do this*) to 4 (*I do this most of the time*). Cronbach’s alphas for the subscales were adequate in the original version (0.54-0.71), the Portuguese adaptation (0.62-0.78), and in the present study (0.65-0.79). All BCI subscales were used as independent variables.

### Statistical analysis

Data were analyzed using IBM SPSS Statistics (v. 25, https://www.ibm.com/). Since skewness and kurtosis values were < 2 and < 4, parametric tests were used. Preliminarily, sociodemographic-clinical groups were compared using Student’s t-tests for independent samples and analyses of variance (ANOVA) to check for differences in BSI-PSDI scores.

For the first objective, Student t-tests or ANOVA with Tukey’s post-hoc tests were calculated to assess differences in means between groups based on gender, age, education level, and psychiatric disorder diagnosis.

For the second objective, Pearson’s correlations were calculated to examine the relationship between continuous variables, specifically neuroticism and agreeableness, coping strategies (denial, self-blame, self-distraction, positive reframing, and behavioral disengagement), and psychological distress (BSI-PSDI).

For the third objective, multiple linear regression analysis (enter method) was computed to identify independent predictors of psychopathological symptoms (BSI-PSDI) after controlling for the role of sociodemographic variables and personality dimensions. Sociodemographic and clinical variables (gender, age, educational qualifications, and psychiatric diagnosis) were entered in Block 1; neuroticism and agreeableness (NEO-FFI) were added in Block 2; and coping strategies (BCI strategies presenting significant correlation values with BSI-PSDI) in Block 3. Assumptions regarding sample size, normality, linearity, multi-collinearity, homoscedasticity, and the inexistence of outliers were confirmed.

Effect sizes were interpreted as: low/small (*r* = 0.10-0.29, *g* = 0.20-0.49, η^2^ = 0.02); moderate/medium (*r* = 0.30-0.49, *g* = 0.50-0.79, η^2^ = 0.13); and high/large (*r* = 0.50-1.0, *g* = 0.80-1.29, η^2^ = 0.26) ^37^.

For most statistical tests, the significance level was set at p < 0.05. The Bonferroni adjustment was used to correct for multiple post-hoc pairwise comparisons (three groups: p < 0.017; four groups: p < 0.0125; six groups: p < 0.003).

## Results

Preliminary analysis showed the global sample BSI scores as follows: somatization (M = 0.65; SD = 0.77), obsession-compulsion (M = 1.12; SD = 0.86), interpersonal sensitivity (M = 0.83; SD = 0.87), depression (M = 0.95; SD = 0.85), anxiety (M = 1.06; SD = 0.86), hostility (M = 0.81; SD = 0.70), phobic anxiety (M = 0.77; SD = 0.88), paranoid ideation (M = 0.96; SD = 0.83), psychoticism (M = 0.69; SD = 0.71), and BSI-PSDI (M = 1.69; SD = 0.57). The global sample had higher mean scores on somatization, depression, anxiety, phobic anxiety, and psychoticism compared to the average reference values of individuals from the general population. Moreover, the global sample’s 1.69 BSI-PSDI score was very close to the 1.71 cut-off point ^32^, indicating a high level of psychological distress. Based on the BSI-PSDI cut-off, 42.7% (n = 1,026) of the sample may have experienced severe psychological distress.

In line with our first objective and hypothesis, results showed that BSI-PSDI scores differed according to various sociodemographic and clinical variables. Specifically, higher levels of psychological distress were observed among women, younger individuals, those with less formal education, those with education and technical professions, and those with a psychiatric diagnosis (Table 1).

Regarding our second objective and hypothesis, Table 2 illustrates Pearson’s correlations across the study variables. All BSI dimensions and BSI-PSDI, ranging from 25% to 46.2%, correlated highly and positively with neuroticism (NEO-FFI) and with most coping strategies (self-distraction, denial, self-blame, behavioral disengagement) and negatively with agreeableness (NEO-FFI) and with positive reframing coping strategy. Of note was the highest positive correlation between the depression subscale (BSI) and neuroticism (*r* ^*2*^ = 46.2%).

**Table 2:** Pearson correlations between psychopathological symptomatology and psychological distress (BSI), Personality (NEO-FFI), and coping strategies (BCI) (N = 2,402).

	1	2	3	4	5	6	7	8	9	10	11	12	13	14	15	16
1	-															
2	0.62	-														
3	0.70	0.68	-													
4	0.60	0.60	0.69	-												
5	0.74	0.65	0.76	0.78	-											
6	0.74	0.80	0.74	0.65	0.76	-										
7	0.64	0.62	0.67	0.67	0.68	0.70	-									
8	0.59	0.59	0.57	0.45	0.52	0.68	0.47	-								
9	0.57	0.56	0.65	0.77	0.70	0.61	0.65	0.43	-							
10	0.68	0.67	0.75	0.79	0.81	0.73	0.72	0.57	0.74	-						
11	0.56	0.50	0.59	0.60	0.68	0.61	0.52	0.40	0.52	0.61	-					
12	-0.21	-0.18	-0.25	-0.33	-0.29	-0.21	-0.36	-0.15	-0.39	-0.34	-0.34	-				
13	0.22	0.14	0.17	0.15	0.18	0.21	0.20	0.15	0.13	0.14	0.21	< 0.10	-			
14	0.18	0.24	0.23	0.21	0.24	0.24	0.24	0.18	0.26	0.24	0.22	-0.15	0.18	-		
15	-0.17	-0.18	-0.22	-0.24	-0.31	-0.24	-0.21	-0.15	-0.19	-0.24	-0.39	0.27	0.14	-0.03	-	
16	0.33	0.21	0.32	0.33	0.34	0.26	0.30	0.13	0.29	0.32	0.34	-0.14	0.23	0.21	< -0.10	-
17	0.31	0.29	0.36	0.37	0.41	0.33	0.33	0.24	0.33	0.38	0.39	-0.25	0.12	0.31	-0.23	0.30

BSI: *Brief Symptom Inventory*: 1 = PSDI: *Positive Symptom Distress Index*; 2 = somatization; 3 = obsession-compulsion; 4 = interpersonal sensitivity; 5 = depression; 6 = anxiety; 7 = hostility; 8 = phobic anxiety; 9 = paranoid ideation; 10 = psychoticism; NEO-FFI: *NEO-Five Factor Inventory*: 11 = neuroticism; 12 = agreeableness; BCI: *Brief-COPE Inventory*: 13 = self-distraction; 14 = denial; 15 = positive reframing; 16 = self-blame; 17 = behavioral disengagement.

Note: values in bold = p < 0.01.

We computed the predictive model of psychopathological symptomatology (BSI-PSDI) using multiple linear regression (enter method) to address our third objective and hypothesis. All variables included in the first block (gender, age, education, and having/not having a psychiatric diagnosis) were shown as predictors, explaining 12% of the total variance, with “having/not having a diagnosed psychiatric disorder” showing the highest predictive value (β = 0.31; p < 0.001). In the second block, after accounting for the previous ones, neuroticism (β = 0.50; p < 0.001) explained 34% of the total variance. Finally, in the third block, 37% of the variance in psychopathological symptomatology was explained by self-distraction, self-blame, and behavioral disengagement, with self-blame being the biggest predictor (β = 0.12; p < 0.001). Finally, agreeableness and positive reframing were not significant predictors of psychopathological symptomatology (Table 3).

**Table 3:** Multiple regression results of psychopathological symptomatology (N = 2,402).

Predictor	r	r^2^	F	p-value *	B	β	t	p-value *
Block 1	0.35	0.12	77.13	< 0.001	2.22	-	25.74	< 0.001
Gender	-	-	-	-	-0.15	-0.08	-4.02	< 0.001
Age	-	-	-	-	-0.11	-0.10	-4.59	< 0.001
Education	-	-	-	-	-0.05	0.04	-2.13	0.033
Psychiatric disorder	-	-	-	-	0.53	0.31	15.28	< 0.001
Block 2	0.58	0.34	368.04	< 0.001	1.17	-	9.79	< 0.001
Neuroticism	-	-	-	-	0.03	0.50	24.89	< 0.001
Agreeableness	-	-	-	-	-0.01	-0.30	-1.53	0.127
Block 3	0.61	0.37	26.45	< 0.001	0.92	-	7.56	< 0.001
Self-distraction	-	-	-	-	0.04	0.09	4.66	< 0.001
Denial	-	-	-	-	0.01	0.01	0.47	0.639
Positive reframing	-	-	-	-	0.02	0.04	1.92	0.055
Self-blame	-	-	-	-	0.05	0.12	6.59	< 0.001
Behavioral disengagement	-	-	-	-	0.04	0.08	4.35	< 0.001

β: standardized regression coefficient, representing the relative strength and direction of the predictor’s relationship with the outcome; B: unstandardized regression coefficient, representing the raw effect of the predictor on the outcome; Block 1: sociodemographic and clinical variables; Block 2*: NEO-Five Factor Inventory*; Block 3: *Brief-COPE Inventory*; F: F-statistic, indicating the overall significance of the regression model; r: multiple correlation coefficient; r²: coefficient of determination, representing the proportion of variance explained by the model; t: t-statistic, testing the significance of individual predictors.

Note: the statistically significant strongest predictors are indicated in bold.

* Representing the significance level of the test.

## Discussion

The COVID-19 pandemic has had profound economic and health consequences. The fear of contagion, uncertainty regarding the financial future, reorganization of daily life, and adoption of new habits have all impacted mental health ^3^. However, the manner and intensity of these repercussions varied based on personal characteristics and predispositions. Therefore, this study aimed to investigate whether personality characteristics and coping strategies predicted psychopathological symptoms during the COVID-19 pandemic.

Our preliminary results showed increased mental health symptoms during the early months of the pandemic, including somatization, depression, anxiety, phobic anxiety, and psychoticism. Notably, 42.7% (n = 1,026) of our sample exhibited severe psychological distress. These findings align with other studies reporting anxiety disorders, depression, post-traumatic stress, sleep disturbances, extreme fear of illness, and negative social behaviors due to perceived contagion risk ^6^ ^,^ ^17^ ^,^ ^38^ ^,^ ^39^. At the pandemic’s onset, the Portuguese population showed moderate to severe symptoms of anxiety (27%) and post-traumatic stress and depression (26%) ^40^, with less stress and greater well-being than other countries. Our findings, however, indicate a higher prevalence of severe psychological distress, possibly due to differences in assessment instruments, methodological variations, media exposure, and sociodemographic factors ^17^ ^,^ ^18^ ^,^ ^41^. These results suggest that the mental health impact of the COVID-19 pandemic is a public health concern that requires comprehensive strategies to address.

Regarding the first objective, our study found that younger adults, women, single individuals, and those with lower education experienced higher distress during the pandemic. This finding confirms our hypothesis that these sociodemographic and clinical variables would predict higher levels of psychological distress. Younger individuals faced significant social and educational disruptions, leading to increased distress ^8^ ^,^ ^9^ ^,^ ^10^ ^,^ ^17^ ^,^ ^18^ ^,^ ^41^. Women, potentially due to increased caregiving responsibilities and greater psychological vulnerability during the pandemic, reported higher levels of distress ^12^ ^,^ ^13^ ^,^ ^17^ ^,^ ^18^ ^,^ ^41^. The social isolation experienced by single individuals and the potential support system available to married individuals may explain the differences in distress levels ^9^ ^,^ ^14^. Those with higher education might have had better access to resources and coping strategies, whereas those with lower educational attainment might have faced greater job insecurity, contributing to their distress ^18^ ^,^ ^41^ ^,^ ^42^.

These sociodemographic factors have significant implications for public health strategies. Targeted mental health support and resources should be developed to mitigate the psychological impact of the pandemic, particularly for younger adults, women, single individuals, and those with lower education.

Interestingly, health and service care workers reported lower distress, which is somewhat surprising given their high-risk exposure and stress. This might be explained by their heightened sense of purpose and strong support structures ^11^. However, other studies noted increased mental health challenges among frontline healthcare workers ^7^ ^,^ ^43^, possibly because our study did not distinguish between frontline workers and other healthcare professionals. Education and technical professionals, including students, teachers, and engineers, reported higher distress, possibly due to unique occupational stressors. Individuals with pre-existing psychiatric disorders reported higher distress, highlighting their heightened vulnerability during the pandemic, as documented in various studies ^14^ ^,^ ^15^ ^,^ ^16^. Public health strategies should aim to strengthen support systems for all essential workers, providing resources and interventions to maintain their mental health during crises.

For the second objective, we found significant correlations between psychopathological symptoms and both personality traits and coping strategies. Specifically, high levels of neuroticism were positively associated with all dimensions of psychological distress and the overall BSI-PSDI. This reinforces the literature suggesting neuroticism is a critical predictor of mental health problems ^44^ ^,^ ^45^. Neuroticism’s strong association with the depression subscale highlights its role in exacerbating depressive symptoms, likely due to the tendency of neurotic individuals to perceive situations negatively and higher emotional reactivity to stress ^46^. This finding underscores the pathological nature of neuroticism, distinguishing it from other personality dimensions that are more adaptive ^47^. Our results also showed that maladaptive coping strategies, such as self-distraction, denial, self-blame, and behavioral disengagement, were positively correlated with psychological distress. These strategies appear ineffective in managing stress, leading to higher levels of psychological symptoms by preventing individuals from addressing the root causes of their distress and by fostering negative emotional states ^35^ ^,^ ^48^. These findings emphasize the need for public health interventions that promote adaptive coping strategies and resilience. Training programs that help individuals develop positive coping mechanisms could mitigate the negative impact of maladaptive strategies. Health professionals should receive specialized training to help individuals develop effective coping strategies, particularly those with high neuroticism levels.

Conversely, we found negative correlations between psychological distress and both agreeableness and positive reframing. Agreeableness, characterized by compassion and cooperativeness, is linked to better social support and lower stress levels ^49^ ^,^ ^50^. Positive reframing, a strategy involving viewing stressors positively, is associated with reduced stress and improved mental health outcomes ^22^ ^,^ ^51^. Thus, these traits and coping strategies may buffer against psychological distress, promoting more resilient responses to stress. Promoting traits such as agreeableness and strategies like positive reframing can be beneficial in public health approaches to mental health. Programs that encourage compassion, cooperation, and positive thinking can enhance social support networks and improve overall mental well-being during crises.

Concerning the third objective, the analysis showed that gender, age, education, and psychiatric diagnosis status predicted 12% of psychopathological symptomatology, with psychiatric diagnosis being the most significant factor. Adding neuroticism increased the explained variance to 34%, indicating its major role in predicting psychopathological distress. Coping strategies, specifically self-distraction, self-blame, and behavioral disengagement, further increased the prediction to 37%, with self-blame being the strongest predictor. These findings confirm our third hypothesis that personality traits and coping strategies are significant predictors of psychological distress, even after controlling for sociodemographic variables. The exception was agreeableness and positive reframing, which were not significant predictors of psychopathological symptomatology.

These findings align with previous research indicating that personality traits, particularly neuroticism, are significant predictors of psychological distress ^20^ ^,^ ^27^ ^,^ ^28^ ^,^ ^43^ ^,^ ^45^ ^,^ ^52^ ^,^ ^53^. Neuroticism’s association with emotional instability likely explains its substantial predictive value, supporting the idea that it is a possible pathological dimension of personality, while the remaining dimensions are deemed positive aspects of personality ^47^. Coping strategies such as self-blame, self-distraction, and behavioral disengagement were positively associated with psychopathological symptoms, suggesting these strategies are ineffective for managing stress. Self-blame, in particular, emerged as the strongest predictor of psychological distress, consistent with a study linking self-blame to increased fear of COVID-19, perceived stress, and depressive symptoms ^54^.

Our findings contrast with some literature emphasizing the protective effects of agreeableness and positive reframing ^22^. Although these traits are generally considered beneficial for mental health, they did not significantly predict psychological distress in our study. This aligns with other studies identifying maladaptive coping strategies as the strongest predictors of emotional outcomes such as anxiety and stress ^28^ ^,^ ^52^ ^,^ ^53^. This discrepancy underscores the need for further research to explore the roles of these traits in different populations and circumstances. These findings suggest that public health interventions should focus on identifying individuals with high levels of neuroticism and providing them with targeted support to prevent psychological distress. Addressing maladaptive coping strategies such as self-blame can further reduce distress levels.

## Limitations

Some limitations in our study need consideration. First, we acknowledge that common method variance could be a factor, given the use of self-response instruments and online administration. This variance may inflate relationships, so future studies should include a measure of social desirability ^55^ or other sources for these constructs ^56^.

Second, other risk factors of psychopathological symptoms were not controlled, including socioeconomic status, having a higher risk of contracting the disease, social isolation, and stressful events related to COVID-19 ^7^. However, by not limiting our focus to specific risk factors, our study provides a broad perspective on psychological distress and coping strategies during the pandemic, offering valuable insights that remain relevant for understanding the long-term mental health impacts of similar global crises.

Third, a limitation of our study was the inability to track the exact number of clicks on the survey link and, consequently, the response rate relative to the number of accesses. Additionally, as our survey distribution relied on organic reach, we do not have precise metrics on the percentage of the targeted population reached, which is a common limitation in studies using social media for recruitment. Despite these limitations, the high level of participation suggests that our approach effectively reached a diverse and substantial audience.

Fourth, our study did not distinguish between frontline health workers and other healthcare professionals. This lack of distinction may affect our understanding of the psychological distress experienced by healthcare workers, as those on the frontline might face different stressors compared to their non-frontline counterparts. However, this differentiation was not the primary objective of our study, which aimed to broadly assess psychological distress and coping strategies during the COVID-19 pandemic.

Fifth, our study is limited by the overrepresentation of individuals with higher education levels (71.8%), compared to the general Portuguese population. According to Organisation for Economic Co-Operation and Development (OECD) data ^57^, only 23.1% hold a Bachelor’s degree, 9.4% a Master’s, and 0.7% a PhD. This discrepancy may impact the generalizability of our findings. Future research should include a more representative sample to enhance external validity.

Sixth, the variable “psychiatric disorder diagnosis” was self-reported and based on the participants’ current diagnosis and treatment status, including taking medication or undergoing therapy at the time of the survey. This approach may have limitations, such as reliance on self-reporting accuracy and the exclusion of past psychiatric disorders that were not currently being treated. Future studies should consider obtaining more detailed psychiatric histories, including lifetime diagnoses, for providing a comprehensive understanding of participants’ mental health.

Finally, although the analyses suggest causal relationships, the cross-sectional nature of our study does not allow us to establish them. Studies, which have since been carried out in the meantime, should assess the impact of COVID-19 at the level of psychopathological symptomatology to understand the pandemic’s future impact and better identify protective (e.g., self-compassion, resilience) and risk variables (e.g., guilt, shame, self-criticism) of the development of psychopathological symptomatology ^58^ ^,^ ^59^ ^,^ ^60^.

## Conclusion

In conclusion, our data provide evidence for a link between psychopathological symptomatology, psychological distress, less protective personality characteristics (such as neuroticism), and maladaptive coping strategies (such as self-distraction, denial, self-blame, and behavioral disengagement). Notably, neuroticism and self-blame were the best predictors of psychological distress. When we also considered maladaptive coping strategies like self-blame, our understanding of what contributes to psychological distress became even clearer, highlighting the complex and multifaceted nature of psychological distress.

Given these findings, promoting resilience and adaptive strategies during prolonged pandemic scenarios is essential. Health professionals should receive specialized training to help individuals develop effective coping strategies, particularly planning, positive reframing, acceptance, and use of emotional support. Additionally, it is essential to increase the availability of mental health resources, focusing on individuals with psychological vulnerabilities, such as high levels of neuroticism.

In this context, one promising approach is Acceptance and Commitment Therapy (ACT), which focuses on accepting what is out of one’s control and committing to actions aligned with personal values. ACT helps individuals identify negative habits and manage difficult thoughts and emotions, fostering a more resilient response to stress ^61^. By integrating these therapeutic approaches, the complex nature of psychological distress can better be addressed, improving mental health outcomes during challenging times like a pandemic.
